# Upregulation of miR-328 and inhibition of CREB-DNA-binding activity are critical for resveratrol-mediated suppression of matrix metalloproteinase-2 and subsequent metastatic ability in human osteosarcomas

**DOI:** 10.18632/oncotarget.3088

**Published:** 2014-12-30

**Authors:** Shun-Fa Yang, Wei-Jiunn Lee, Peng Tan, Chih-Hsin Tang, Michael Hsiao, Feng-Koo Hsieh, Ming-Hsien Chien

**Affiliations:** ^1^ Institute of Medicine, Chung Shan Medical University, Taichung, Taiwan; ^2^ Department of Medical Research, Chung Shan Medical University Hospital, Taichung, Taiwan; ^3^ Department of Urology, Wan Fang Hospital, Taipei Medical University, Taipei, Taiwan; ^4^ Graduate Institute of Clinical Medicine, College of Medicine, Taipei Medical University, Taipei, Taiwan; ^5^ Graduate Institute of Basic Medical Science, China Medical University, Taichung, Taiwan; ^6^ The Genomics Research Center, Academia Sinica, Taipei, Taiwan; ^7^ Experimental Surgery and Regenerative Medicine, Department of Surgery, Ludwig-Maximilians University, Munich, Germany; ^8^ Department of Medical Education and Research, Wan Fang Hospital, Taipei Medical University, Taipei, Taiwan

**Keywords:** osteosarcoma, miR-328, CREB, MMP-2, resveratrol, metastasis

## Abstract

Osteosarcomas, the most common malignant bone tumors, show a potent capacity for local invasion and pulmonary metastasis. Resveratrol (RESV), a phytochemical, exhibits multiple tumor-suppressing activities and has been tested in clinical trials. However, the antitumor activities of RESV in osteosarcomas are not yet completely defined. In osteosarcoma cells, we found that RESV inhibited the migration/invasion *in vitro* and lung metastasis *in vivo* by suppressing matrix metalloproteinase (MMP)-2. We identified that RESV exhibited a transcriptional inhibitory effect on MMP-2 through reducing CREB-DNA-binding activity. Moreover, a microRNA (miR) analysis showed that miR-328 was predominantly upregulated after RESV treatment. Inhibition of miR-328 significantly relieved MMP-2 and motility suppression imposed by RESV treatment. Furthermore, ectopic miR-328 expression in highly invasive cells decreased MMP-2 expression and invasive abilities. Mechanistic investigations found that JNK and p38 MAPK signaling pathways were involved in RESV-regulated CREB-DNA-binding activity, miR328 expression, and cell motility. Clinical samples indicated inverse expression between MMP-2 and miR-328 in normal bone and osteosarcoma tissues. The inverse correlation of MMP-2 and miR-328 was also observed in tumor specimens, and MMP-2 expression was linked to tumor metastasis. Taken together, our results provide new insights into the role of RESV-induced molecular and epigenetic regulation in suppressing tumor metastasis.

## INTRODUCTION

Osteosarcomas are highly aggressive malignant tumors of the bone that rank as the main cause of cancer-related deaths in adolescents and young adults. At present, modern treatment protocols that combine chemotherapy, surgery, and occasionally radiotherapy can augment the 5-year survival rate of patients with an osteosarcoma by around 60%~70%, if the disease is localized [1, 2]. However, a majority of patients with this bone tumor may harbor “micrometastasis“ at the time of diagnosis, and many develop multidrug resistance during treatment [3]. Osteosarcoma patients with metastasis have a poor prognosis, and the long-term survival rate remains at 10%~30% [4]. It seems difficult to improve current response rates with further dose escalations to overcome drug resistance, as resistant tumor cells are able to withstand the effects of cytotoxic agents. In addition, associated cytotoxic side effects on normal tissues and organs remain a serious drawback. Therefore, there is a pressing need to develop new and alternative approaches to the current medical treatment of osteosarcomas. In this regard, dietary supplements and phytotherapeutic agents with high anticancer efficacy and nominal toxicity to normal tissues are suggested as possible candidates. For example, the successful use of two representative drugs, paclitaxol, extracted from *Taxus brevifolia,* and vinorelbine, derived from the periwinkle plant, was reported to have successfully treated several clinical cancers [5, 6].

Metastasis of cancer cells involves multiple processes and various cytophysiological changes, including changing the adhesion capabilities between cells and the extracellular matrix (ECM) and disrupting intercellular interactions. Degradation and dissociation of the ECM are also needed for metastasis of osteosarcomas to occur [7]. Thus, degradation of the ECM and components of the basement membrane caused by the concerted action of proteinases, such as matrix metalloproteinases (MMPs), cathepsins, and the plasminogen activator (PA), play a critical role in tumor invasion and metastasis [8, 9]. MMPs are overexpressed in almost all human cancers including osteosarcomas [10-12] and are thought to be a promising therapeutic target for osteosarcoma patients [13]. Of the MMPs, MMP-2, MMP-9, and their upstream enzyme, urokinase-PA (u-PA), are the most vital enzymes for degrading the main constituent of the basement membrane, type IV collagen, and are deeply involved in cancer invasion and metastasis [14]. Therefore, inhibiting the migration or invasion mediated by MMP-2, MMP-9, or u-PA could putatively provide a preventive measure against cancer metastasis.

Micro (mi)RNAs are small, endogenous, 21~23-nucleotide-noncoding RNAs that belong to a novel class of gene regulators with critical roles in physiologic and pathologic processes including development, viral infection, and cancer [15, 16]. Functional characterization showed that miRNAs function as oncogenes or tumor-suppressor genes through respectively binding to 3′ untranslated regions (UTRs) of target tumor suppressor genes or oncogenic genes. For example, increasing numbers of miRNAs have been reported to regulate MMPs [17], and the array-based miRNA profiling of human cancer cells has identified an association between miRNA deregulation and cancer metastasis [18, 19]. Until now, our understanding of miRNA-related mechanisms in tumor metastasis has been incomplete, and they remain to be investigated and characterized.

Resveratrol (3,5,4′-trihydroxystilbene, RESV) is a natural polyphenol present in various plants, including grapes, berries, and peanuts [20]. For cancer therapy, RESV reportedly inhibits cancer progression at the initiation, promotion, and progression steps, and it also has chemoprevention abilities by inhibiting or activating molecular targets such as kinases, cyclooxygenases, ribonucleotide reductase, DNA polymerases, and Sirt1 [21-25]. Moreover, RESV inhibits several transcriptional factors, including nuclear factor (NF)-κB, activator protein (AP)-1, AP-2, and cAMP response element-binding (CREB), which act independently or in coordination to regulate many genes involved in regulating u-PA and MMPs [26, 27]. RESV was reported to exhibit anticancer properties in various cancer cells, including breast, prostate, stomach, colon, pancreas, and thyroid cancers [28, 29] and was recently used in phase I clinical trials for colon cancer [30]. However, compared to other tumor types, data regarding the antimetastatic effects of RESV on osteosarcomas are scarce. Thus, in the present study, we investigated the effects of RESV on the cell motility of osteosarcoma cells and elucidated the possible underlying mechanisms. The results showed that RESV suppresses the metastatic potential of human osteosarcomas through transcriptional and epigenetic regulation of MMP-2 by respectively inhibiting CREB-DNA-binding activity and upregulating miR-328.

## RESULTS

### RESV inhibits the cell migration, invasion, and adhesive abilities of human osteosarcoma cells

**Figure 1:**
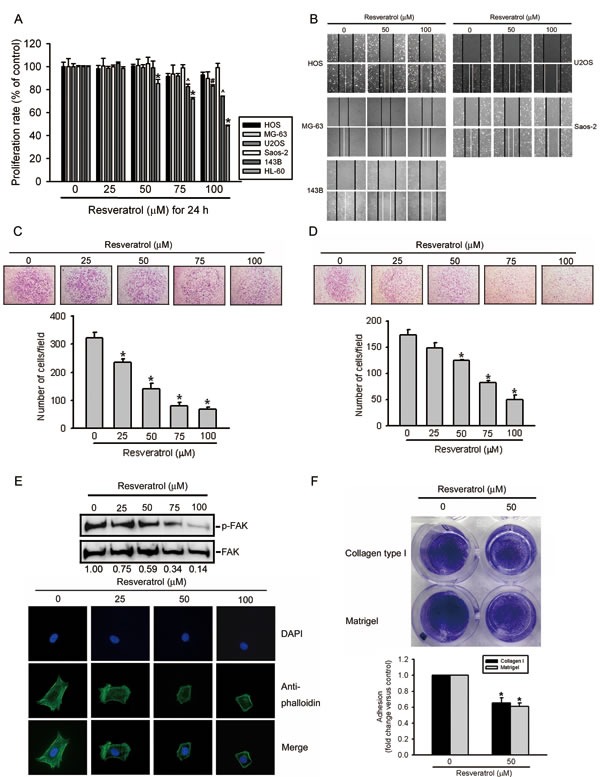
Resveratrol inhibits cell migration, invasion, and adhesion abilities of human osteosarcoma cells (A) Effect of resveratrol on cell viability of osteosarcoma cells. Five osteosarcoma cell lines were treated with the vehicle or resveratrol (25~100 μM) in serum-containing medium for 24 h. Cell viability was determined by an MTS assay. HL-60 cells are used for a positive control after resveratrol treatment. Values represent the mean ± SE of three independent experiments. ^(*, #, ^)^ *p* < 0.05, compared to the vehicle groups. (B) Effect of resveratrol on wound closure in osteosarcoma cells. Five osteosarcoma cell lines were wounded and then treated with vehicle or resveratrol (50~100 μM) for 24 h, and phase-contrast pictures of the wounds were taken. (C, D) HOS cells were treated by the indicated concentrations of resveratrol for the transwell migration and Matrigel invasion assays. Values are presented as the mean ± SE of three independent experiments. * *p* < 0.05, compared to the vehicle groups. (E) Effect of resveratrol on cellular morphology and FAK activation. HOS cells were treated with different concentration of resveratrol or its control, as indicated, for 24 h. Upper panel, FAK activation was examined by Western blotting. Quantitative p-FAK protein levels were adjusted to the total FAK protein level. Lower panel, Cells were fixed and stained for F-actin by Alexa Fluor® 488-conjugated phalloidin (green). Nuclei were counterstained with DAPI (blue). (F) Effect of resveratrol on the cell adhesion ability using Matrigel or collagen type I. HOS cells in wells that were coated with Matrigel or collagen type I were pretreated with 50 μM resveratrol or vehicle for 24 h. After a 30-min incubation, numbers of adhering cells were determined by an MTT assay at 570 nm. Values are presented as the mean ± SE of three independent experiments. * *p* < 0.05, compared to the vehicle groups.

It was recently reported that long-term treatment (3~7 days) with RESV can inhibit the growth of human osteosarcoma cell lines [31]. To further investigate the pharmacological potential of RESV against osteosarcomas, we first examined the effects of short-term treatment (24 h) with RESV on cell migration, invasion, and adhesion in osteosarcoma cells. The cytotoxic effects of RESV treatment for 24 h at various concentrations (0~100 μM) on five osteosarcoma cell lines (HOS, MG-63, U2OS, Saos-2, and 143B) are shown in Figure 1A. An MTS assay showed that even at the highest concentration of 100 μM, RESV only partially altered or did not alter the viability of these osteosarcoma cell lines with 24-h treatment, compared to that of the controls. The leukemic cell line, HL-60, was used as a positive control after RESV treatment (Figure 1A). To further determine the roles of RESV in cell migration and invasion, five highly invasive osteosarcoma cell lines, HOS, MG-63, U2OS, Saos-2, and 143B, were treated with various concentrations of RESV for 24 h. Figure 1B contains photographs of HOS, MG-63, U2OS, Saos-2, and 143B cells migrating into scratch wounds, and showed that RESV concentration-dependently suppressed wound closure in these five osteosarcoma cell lines. A Boyden chamber assay was further used to investigate the effects of RESV on the migration and invasion of HOS cells, and results showed that RESV also had a significant inhibitory effect on cell migration and invasion at concentrations of 25~100 μM (Figure 1C, D). To further assess whether RESV suppresses cell motility via arrangements of the actin cytoskeleton, HOS cells treated with RESV were stained with an FITC-conjugated anti-phalloidin antibody and characterized using immunofluorescence microscopy. HOS control cells displayed well-formed F-actin-containing microfilament bundles within the cytoplasm, whereas cells treated with RESV contained few microfilament bundles (Figure 1E, lower panel). The increase in tyrosine phosphorylation in focal adhesion kinase (p125-FAK) is accompanied by profound alterations in the organization of the actin cytoskeleton, and our results also showed that RESV concentration-dependently attenuated the tyrosine phosphorylation of p125-FAK in HOS cells (Figure 1E, upper panel). These findings indicated that RESV regulates the rearrangement of F-actin-containing microfilament bundles, suggesting that rearrangement of F-actin-containing microfilament bundles may be involved in RESV-regulated cell invasiveness. Because the adhesion and motility of tumor cells in the ECM are considered important steps in the invasive process of metastatic tumor cells, the effects of RESV on cell adhesion were further examined. Incubation of HOS cells with 50 μM RESV for 24 h significantly inhibited cell adhesion to Matrigel- and collagen-coated substrates (Figure 1F). According to these data, RESV significantly inhibited cancer cell invasion at a non- or low-cytotoxic concentration, indicating that RESV is an effective inhibitor of cell motility and adhesion in osteosarcoma cells.

### RESV attenuates uPA-mediated MMP-2 activation and causes transcriptional suppression of the *MMP-2* gene

**Figure 2:**
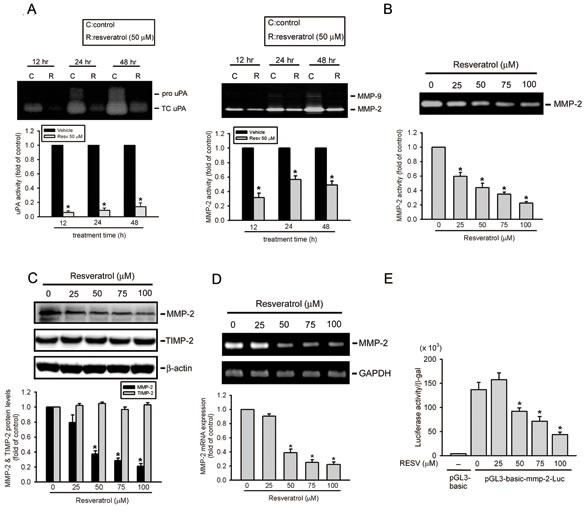
Resveratrol attenuates urokinase plasminogen activator (uPA)-mediated matrix metalloproteinase (MMP)-2 activation and represents transcriptional suppression of the MMP-2 gene (A, B) Effects of resveratrol on the activities of u-PA and MMP-2/9. HOS cells were treated with 50 μM resveratrol for the indicated time points or different concentrations of resveratrol for 24 h and then subjected to zymography to analyze the activities of uPA (Figure 2A, left panel) and MMP-2/9 (Figures 2A, right panel; 2B). (C) Effects of resveratrol on the protein level of MMP-2 and its endogenous inhibitor, TIMP-2. HOS cells were treated with the vehicle or resveratrol (25~100 μM) for 24 h and then subjected to a Western blot analysis. Quantitative MMP-2 and TIMP-2 protein levels were adjusted to the β-actin protein level. (D, E) HOS cells were treated with resveratrol (25~100 μM) for 24 h and then subjected to an RT-PCR to analyze mRNA expression of MMP-2 (Figure 2D), or an MMP-2 promoter reporter assay to analyze the promoter activity of MMP-2 (Figure 2E). Luciferase activity, determined in triplicate, was normalized to β-galactosidase activity. Values are presented as the mean ± SE of three independent experiments. * *p* < 0.05, compared to the vehicle groups.

The epithelial-mesenchymal transition (EMT) and degradation of the ECM were shown to play important roles in regulating the invasion and migration of osteosarcoma cells [7, 32]. Effects of RESV on the EMT and ECM degradation of osteosarcoma cells were examined by treating HOS cells with RESV for the indicated time points (12~48 h) and observing EMT- and ECM degradation-related proteins. We found that 50 μM of RESV inhibited the proteolytic activity of MMP-2 and its upstream activator, uPA, at different time points (Figure 2A), but had no effect on the EMT-related marker, E-cadherin (data not shown). Moreover, after treatment of HOS cells with different concentration of RESV (25~100 μM) for 24 h, MMP-2 enzyme activity was concentration-dependently suppressed by RESV (Figure 2B). These data indicate that decreased cell motility after RESV treatment may be due to inhibition of uPA-mediated MMP-2 activation.

In addition to enzyme activity, the protein (Figure 2C) and mRNA (Figure 2D) levels of MMP-2 were also suppressed by RESV in concentration-dependent manners, whereas the endogenous inhibitor of MMP-2, TIMP-2, was not affected by RESV (Figure 2C). In addition to HOS cells, MMP-2 protein and enzyme activity were also concentration-dependently suppressed by RESV in two other osteosarcoma cell lines, 143B and U2OS, suggesting that suppression of MMP-2 by RESV may be a general phenomenon in osteosarcoma (Supplementary Figure S1). Since RESV inhibited the mRNA expression of MMP-2, we examined whether RESV affected the promoter activity of MMP-2. A luciferase reporter gene containing the MMP-2 promoter region was transiently transfected into HOS cells, and luciferase activity was determined. As shown in Figure 2E, RESV suppressed the promoter activity of MMP-2 in a concentration-dependent manner indicating that RESV regulates the expression of MMP-2, at least partially, at the transcriptional level.

### Significant antimetastatic and antiproliferative effects of RESV in an HOS orthotopic graft model

**Figure 3:**
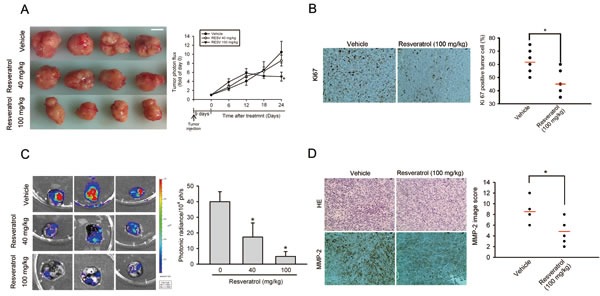
Significant antimetastatic and antiproliferative effects of resveratrol in an HOS orthotopic graft model Luciferase-tagged MNNG/HOS cells were injected into the proximal tibia of SCID mice (seven mice in each group). Nine days after tumor cell injection, the mice were orally fed resveratrol (40 or 100 mg/kg) or vehicle five times per week. The tumor size was monitored by bioluminescence imaging at the indicated time intervals. Twenty-four days after resveratrol treatment, animals were sacrificed, and tumor specimens were collected. (A) Left panel, Gross appearance of orthotopic tumors after treatment with vehicle or resveratrol for 24 days. Scale bar = 1 cm. Right panel, Average tumor volume of each group is shown. (B) A proliferation index was determined based on Ki67 immunostaining, and Ki67-positive cells were counted at ×200 magnification per HOS tumor section (left panel). The mean value of the Ki67 expression percentage is indicated by a red bar. * *p* < 0.05, compared to the vehicle groups (right panel). (C) Lung metastasis was bioluminescently imaged at the end of the study (left panel) with the mean signal for each group (*n*=7) indicated (right panel) * *p* < 0.05, compared to the vehicle groups. (D) Tumor tissues were examined by H&E staining, and immunohistochemical staining with an anti-MMP-2 antibody (left panel). The mean MMP-2 image score is indicated by the red bar (right panel). * *p* < 0.05, compared to the vehicle groups.

To evaluate the antitumor activities of RESV on primary tumor growth and lung metastasis, luciferase-expressing MNNG/HOS-Luc cells were established. Cells were injected into the proximal tibia of severe combined immunodeficient (SCID) mice and allowed to become established for 9 days before initiation of treatment. MNNG/HOS-Luc orthotopic graft mice were treated with different dosages of RESV or the vehicle control five times per week using oral gavage, and tumor growth and metastasis were monitored by bioluminescence imaging. Figure 3A shows the inhibitory potency of RESV on tumor growth after 24 days of treatment by photon emission detection *in vivo*. In RESV-treated mice receiving 100 but not 40 mg/kg, the mean tumor volume on day 24 was significantly inhibited compared to vehicle-treated MNNG/HOS tumors (Figure 3A). Mice were sacrificed at the end of the experiment (33 days after cell injection), and *ex vivo* imaging of the lungs from mice showed a lower intensity in 40- and 100-mg/kg RESV-treated mice compared to vehicle-treated mice (Figure 3C). An IHC analysis of cell proliferation was performed using Ki67 staining. Ki67-positive tumor cells were significantly reduced after treatment with RESV compared to control mice (Figure 3B). Additionally, the expression of MMP-2 in tumor specimens starkly decreased in the RESV-treated group compared to the control group (Figure 3D. These observations suggest that RESV, through suppressing MMP-2, may inhibit cell motility *in vitro* and cell metastasis *in vivo*.

### RESV inhibits the transcriptional activity of MMP-2 by suppressing the DNA-binding activity of CREB on the *MMP-2* promoter

**Figure 4:**
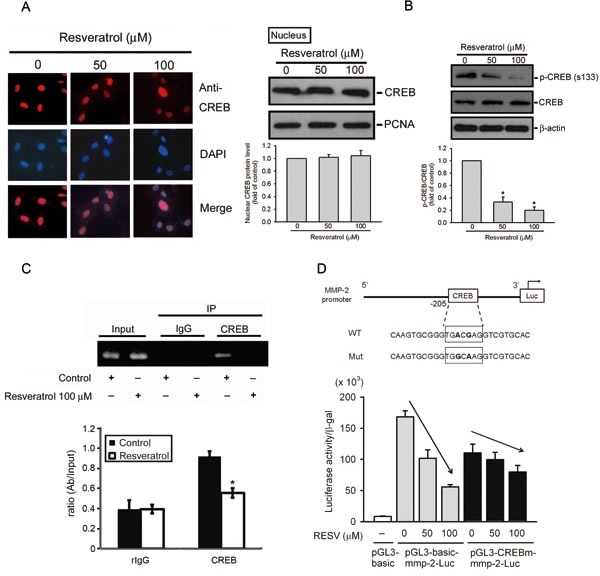
Resveratrol inhibits the transcriptional activity of matrix metalloproteinase (MMP)-2 by suppressing the DNA-binding activity of CREB on the MMP-2 promoter (A, B) Effect of resveratrol on CREB nuclear translocation and phosphorylation. HOS cells were treated with resveratrol for 24 h, and then the nuclear fraction or total cell lysates were prepared and subjected to a Western blot analysis with CREB (Figure 4A, right panel) or p-CREB (Figure 4B) antibodies. Quantitative results of CREB and p-CREB protein levels, which were adjusted to the PCNA or β-actin protein level. Values are presented as the mean ± SE of three independent experiments. * *p* < 0.05, compared to the vehicle groups. The effect of resveratrol on CREB nuclear translocation was confirmed by immunofluorescence (Figure 4A, left panel). Original magnification, 400×. (C) ChIP analysis of the association of the transcription factor, CREB, with the *MMP-2* promoter region in HOS cells. Upper panel, ChIP assays were conducted on HOS cells using a CREB antibody to screen the CREB-bound MMP-2 promoter region for PCR amplification. Lower panel, The effect of resveratrol on the DNA-binding activity of CREB was confirmed by a ChIP-qPCR assay. IgG was used as a negative control. Values are presented as the mean ± SE of three independent experiments. * *p* < 0.05, compared to the vehicle groups. (D) Upper panel, Schematic of the promoter region of the human MMP-2 gene and the utilized wild-type and mutant constructs. Lower panel, HOS cells were co-transfected with pGL3-basic (vector) or MMP-2 promoter constructs (wild-type or CREB-mut) and the pSV-β-galactosidase control vector. Transfected cells were treated with the indicated concentrations of resveratrol for 24 h. Luciferase activity, determined in triplicate, was normalized to β-galactosidase activity. Values are presented as the means ± SE of three independent experiments.

Previous studies indicated that the transcription factor, CREB, plays an important role in controlling MMP-2 expression in various cancer cell lines [33, 34]. Nuclear translocation and phosphorylation of Ser133 are required for CREB-induced gene transcription [35]. Immunofluorescent staining (Figure 4A, left panel) and Western blot results (Figure 4A, right panel) show that RESV did not affect the nuclear localization but inhibited Ser133 phosphorylation of CREB (Figure 4B) in a concentration-dependent manner in HOS cells. We further performed a ChIP assay to investigate the transcription factor's involvement in the transcriptional inhibitory effects of RESV on MMP-2. As illustrated in the upper panel of Figure 4C, binding of CREB to the MMP-2 promoter decreased in HOS cells after RESV treatment. This result was further confirmed by a quantitative real-time PCR assay; RESV indeed significantly suppressed binding of CREB to the MMP-2 promoter (Figure 4C, lower panel). To further determine if the transcription factor, CREB, participates in regulation of MMP-2 transcription, we generated a promoter with a mutation in the CREB-binding site. Results showed that the inhibitory potential of RESV against MMP-2 promoter activity was significantly reversed when the CREB-binding site was mutated (Figure 4D). These results indicate that the CREB transcription factor and CREB-binding site in the MMP-2 promoter region might contribute, at least in part, to the inhibitory effect of RESV against MMP-2 transcription.

### The p38 and JNK pathways are involved in RESV-mediated suppression of MMP-2 activity and expression, and cell motility

**Figure 5:**
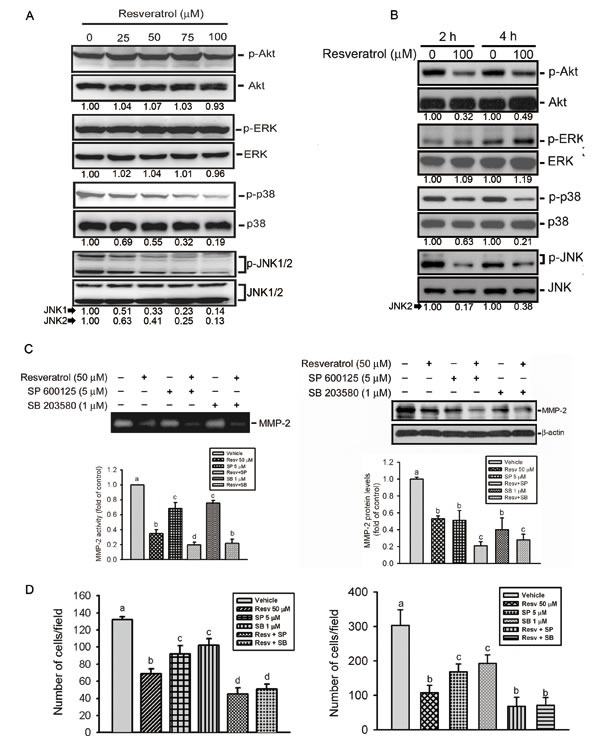
The p38 and c-Jun n-terminal kinase (JNK) pathways are involved in resveratrol-mediated suppression of matrix metalloproteinase (MMP)-2 activity, expression, and cell motility (A, B) Phosphorylation levels of Akt, extracellular signal-regulated kinase (ERK)1/2, p38, and JNK1/2 were assessed by a Western blot analysis after treatment of HOS cells with various concentrations of resveratrol (0~100 μM) for 24 h (A) or 100 μM resveratrol for the indicated time points (B). Quantitative results of p-Akt, p-ERK, p-p38, and p-JNK1/2 protein levels, which were respectively adjusted to their total protein levels. (C) HOS cells were pretreated with or without SP600125 (5 μM) or SB203580 (1 μM) for 1 h followed by resveratrol (50 μM) treatment for an additional 24 h. MMP-2 activity (left panel) and expression level (right panel) were respectively determined by zymography and Western blot analyses. Quantitative MMP-2 protein levels were adjusted to the β-actin protein level. (D) HOS cells were pretreated with or without SP600125 (5 μM) or SB203580 (1 μM) for 1 h followed by resveratrol (50 μM) treatment for an additional 24 h. Cell migration (left panel) and invasion (right panel) abilities were respectively determined by wound-closure and Matrigel invasion assays. Values are presented as the mean ± SE of three independent experiments. Data were analyzed using a one-way ANOVA with Tukey's post-hoc tests at 95% confidence intervals; different letters represent different levels of significance.

The Akt and MAPK pathways were reported to regulate MMP expressions and cancer metastasis [36]. We therefore examined whether the Akt and MAPK pathways are involved in RESV-mediated suppression of cell motility and MMP-2 expression in osteosarcoma cells. Our results show that treatment of HOS cells with RESV for 24 h suppressed activation of JNK1/2 and p38 MAPK in dose-dependent manners (Figure 5A). Moreover, after treatment of HOS cells with RESV for 2~4 h, inhibition of Akt activation was also observed (Figure 5B). Next, we further investigated relationships among RESV-mediated inhibitory effects on MMP-2, cell motility, and MAPKs. Pretreatment of HOS cells with a p38 MAPK inhibitor (SB 203580) or a JNK inhibitor (SP 600125) enhanced the inhibitory effect of RESV against MMP-2 activity (Figure 5C, left panel), MMP-2 expression (Figure 5C, right panel), and cell migration (Figure 5D, left panel) and invasion (Figure 5D, right panel), indicating that the p38 and JNK pathways might be involved in RESV-mediated suppression of MMP-2 activity and expression, and cell motility.

### Upregulation of miR-328 is involved in RESV-mediated suppression of MMP-2 expression and cell motility

**Figure 6:**
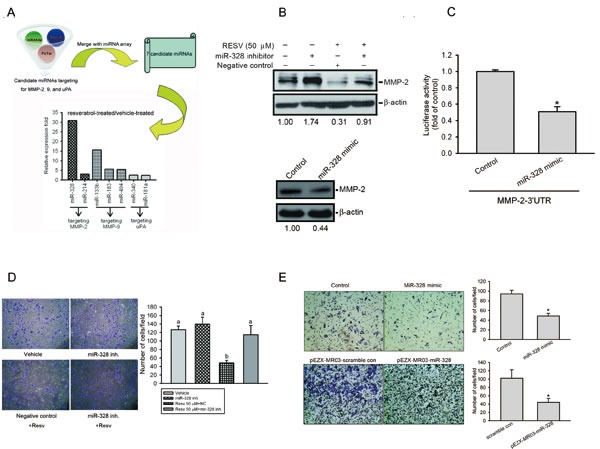
Upregulation of miR-328 is involved in resveratrol-mediated suppression of matrix metalloproteinase (MMP)-2 expression and cell motility (A) A schematic representation of the procedure for miRNA selection. Differential expressions of miRNAs in resveratrol-treated cells versus vehicle-treated cells were analyzed by an miRNA microarray analysis. (B) Upper panel, HOS cells were transfected with an miR-328 inhibitor or negative control for 24 h followed by resveratrol (50 μM) treatment for an additional 24 h. Lower panel, HOS cells were transfected with an miR-328 mimic or mimic control for 24 h. The MMP-2 expression level was determined by a Western blot analysis. Quantitative MMP-2 protein levels were adjusted to the β-actin protein level. (C) HOS cells were co-transfected with an MMP-2 luciferase 3′UTR reporter vector and miR-328 mimic or mimic control for 24 h, and the relative luciferase activity was measured. (D) HOS cells were transfected with an miR-328 inhibitor or negative control for 24 h followed by resveratrol (50 μM) treatment for an additional 24 h. The cell invasion ability was determined by a Matrigel invasion assay. Values are presented as the mean ± SE of three independent experiments. Data were analyzed using a one-way ANOVA with Tukey's post-hoc tests at 95% confidence intervals; different letters represent different levels of significance. (E) HOS cells were transfected with either an miR-328 mimic, pEZX-MR03-miR-328, or their respective controls, as indicated. The cell invasion ability was determined by a Matrigel invasion assay. Values are presented as the mean ± SE of three independent experiments. * *p* < 0.05, compared to the control groups.

miRNA was reported to be an important regulator of cancer progression and metastasis [37]. In addition to determining the transcriptional regulation of MMP-2 by RESV, we next investigated whether miRNA participates in RESV-mediated suppression of MMP-2 and cell motility. In order to investigate miRNA differential expression in RESV-treated osteosarcoma cells, we used TaqMan Array Human MicroRNA panels A and B that contained 754 human miRNAs. After treating osteosarcoma cells with RESV for 6 h, *MMP-2* mRNA was significantly downregulated (Supplementary Figure S2A) and the top 20 up- or downregulated miRNAs are shown in Supplementary Figure S2B. To further identify which miRNAs from Supplementary Figure S2B might target MMP-2, MMP-9, or uPA, we searched for possible miRNAs using a bioinformative screening analysis of miRNA target databases: PicTar, miRanda, and Targetscan. As shown in Figure 6A, we found that miR-328 was the most highly upregulated miRNA in response to RESV treatment and might target MMP-2. We confirmed miR-328 expression using a qRT-PCR, and miR-328 was significantly upregulated after RESV treatment for 6 h in HOS cells (Supplementary Figure S2C). Furthermore, the endogenous MMP-2 level in HOS cells was respectively up- or downregulated after transfection with an miR-328 inhibitor (Figure 6B, upper panel) or miR-328 mimic (Figure 6B, lower panel). Transfection of the miR-328 inhibitor significantly reversed the inhibitory effect of RESV on MMP-2 expression (Figure 6B, upper panel). Next, to examine whether miR-328 regulates the 3′UTR of MMP-2, we constructed a luciferase reporter vector harboring the 3′UTR of MMP-2 and transfected this vector combined with the miR-328 mimic or negative control into HOS cells. Results showed that the miR-328 mimic, but not the negative control, decreased luciferase activity (Figure 6C). Taken together, these data demonstrated that miR-328 can be induced by RESV in HOS cells and directly represses MMP-2 protein expression through binding to the 3′UTR of the human *MMP-2* gene. Induction of miR-328 by RESV was also observed in two other osteosarcoma cell lines, 143B and U2OS (Supplementary Figure S3).

To further verify the direct effect of miR-328 on cell motility, we transiently transfected the miR-328 inhibitor into HOS cells, and found that the miR-328 inhibitor significantly reversed RESV-mediated inhibition of cell invasion (Figure 6D) and migration (Supplementary Figure 4A). In contrast, when we transiently (Figure 6E, upper panel) or stably (Figure 6E, lower panel) overexpressed miR-328 into HOS cells, the invasive ability was significantly downregulated compared to control cells, and a similar phenomenon was observed in two other osteosarcoma cell lines, 143B and U2OS (Supplementary Figure S4B). Taken together, our results indicate that RESV suppresses osteosarcoma motility through upregulating miR-328.

### The p38 and JNK pathways are involved in RESV-induced upregulation of miR-328 and suppression of CREB-binding activity of the *MMP-2* promoter

**Figure 7:**
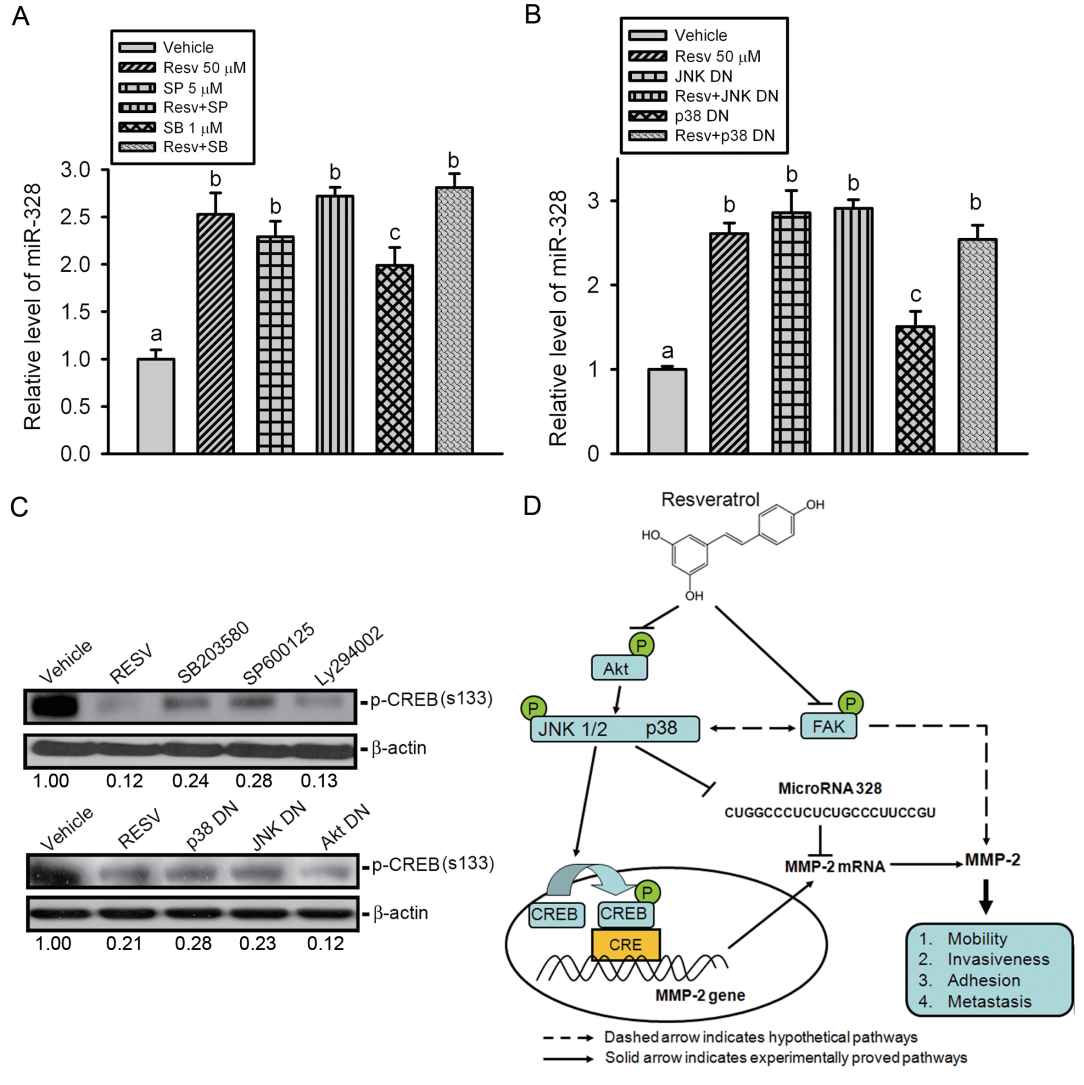
The p38 and c-Jun N-terminal kinase (JNK) pathways are involved in resveratrol-induced upregulation of miR-328 and suppression of CREB phosphorylation (A-C) HOS cells were pretreated with SP600125 (5 μM), SB203580 (1 μM), or Ly294002 (20 μM) for 1 h (A, C) or transfected with a dominant negative mutant (DN) of JNK, p38, or Akt for 24 h (B, C) followed by resveratrol (50 μM) treatment for an additional 24 h. miR-328 expression was examined by a qRT-PCR, and CREB phosphorylation was assayed by a Western blot analysis. Quantitative results of the p-CREB protein level, which was adjusted with the β-actin protein level. Values are presented as the mean ± SE of three independent experiments. Data were analyzed using a one-way ANOVA with Tukey's post-hoc tests at 95% confidence intervals; different letters represent different levels of significance. (D) A working model shows the molecular mechanism underlying the ability of resveratrol to suppress the motility of osteosarcomas.

To further ascertain the role of MAPK pathways in RESV-mediated miR-328 upregulation and CREB-binding activity suppression, HOS cells were pretreated with a JNK or p38 MAPK inhibitor or transfected with their DN mutants followed by RESV treatment. The qRT-PCR data showed that JNK and p38 MAPK inhibitors or mutants only induced miR-328 expression, and a combination of inhibitors or mutants with RESV further enhanced miR-328 upregulation (Figure 7A, B). Moreover, results from the Western blot showed that inhibition of JNK or p38 MAPK by their chemical inhibitors or DN mutants attenuated Ser133 phosphorylation of CREB (Figure 7C). Taken together, our results indicated that p38 MAPK and JNK signaling might play an important role in upregulating RESV-mediated miR-328 and suppressing CREB-binding activity.

### Clinical significance of MMP-2 and miR-328 in osteosarcomas

**Figure 8:**
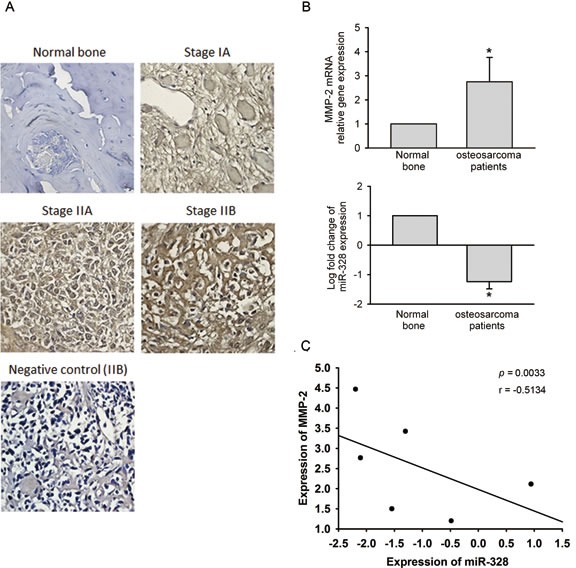
Clinical significance of matrix metalloproteinase (MMP)-2 and miR-328 in osteosarcomas (A) Immunohistochemical staining of MMP-2 in normal bone versus osteosarcoma tissues. (B) To compare expression levels of *MMP-2* mRNA and miR-328 between normal bone and osteosarcoma tissues. Values are presented as the mean ± SE. * *p* < 0.05, compared to the normal control groups. (C) The correlation of MMP-2 and miR-328 expression in osteosarcoma specimens. Pearson's correlation coefficient = −0.5134; *p* = 0.033.

Finally, we investigated the clinical importance of MMP-2 and miR-328 in osteosarcoma patients. Results from IHC staining showed that the expression of MMP-2 in osteosarcoma patients was significantly higher than in normal bone tissues (Figure 8A). The MMP-2 expression level in osteosarcomas was significantly associated with the T stage and metastasis (Table 1). A qRT-PCR analysis of MMP2 and miR-328 performed in osteosarcoma and normal samples, showed inverse expression levels of MMP2 and miR-328 between osteosarcoma and normal bone tissues (Figure 8B), and a significant negative correlation between MMP-2 and miR-328 was observed in osteosarcomas (Figure 8C). These results implied that lower miR-328 expression was linked to higher MMP-2 expression and was correlated with osteosarcoma development and metastasis.

**Table 1:** Clinicopathologic characteristics of patients with associated expression of MMP-2 protein in osteosarcoma

Characteristic	Low expression (n=14)	High expression (n=36)	p-value
Age (year)			
< 24	10	11	0.0043^a^
> 25	4	25	
Gender			
Female	3	14	0.121^a^
Male	11	22	
Stage^b^			
IA	4	3	0.1085^a^
IIA	5	10	
IIB	5	16	
IVB	0	7	
Tumor status^b^			
T1	8	10	0.026^a^
T2-T3	6	26	
Metastasis^b^			
M0	14	29	0.0316^a^
M1	0	7	

a p values were derived with Pearson chi-square tests.

b The tumor stage, tumor status, and metastasis were classified according to the international system for staging osteosarcoma.

## DISCUSSION

Cancer metastasis, the major cause of cancer-related deaths, occurs through a complex multistep process including the intravasation, migration to distant organs, adhesion to endothelial cells lining the blood vessels, and extravasation, and causes the greatest challenge to cancer treatment [38]. Similar to other solid tumor types, primary osteosarcoma management may be successful in most patients, but the development of metastasis to the lungs or other organs produces a high death rate [1]. At present, in addition to chemo drugs, identifying novel approaches to chemoprevention using non-toxic natural substances is of great interest and has been widely investigated. The chemopreventive and antitumor effects of RESV, a natural polyphenol compound, were documented from *in vivo* and clinical studies in a wide variety of tumor cell types [20, 39, 40]. However, compared to other tumor types, data regarding the antimetastatic effects of RESV on osteosarcomas are not yet well defined. This study characterized the antimetastatic effect of RESV on the inhibition of MMP-2 in human osteosarcomas, which is responsible for subsequent decreased migration, adhesion, and metastasis. In addition, this study also identified novel mechanisms of RESV that inhibit MMP-2 through an underlying pathway of suppressing JNK/p38MAPK signals that consequently attenuates CREB-DNA-binding activity and upregulates miR-328 expression. This study provides evidence that RESV treatment in osteosarcoma functionally regulates MMP and substantially inhibits metastasis.

The critical step in tumor invasion and metastasis is reportedly the breakdown of the ECM, which requires activation of proteolytic enzymes [41] such as MMPs, particularly MMP-2 and MMP-9, as elevated levels of these are well documented to indicate an invasive phenotype [42]. Interestingly, a previous report indicated that all osteosarcoma cells from tissue specimens and established cell lines were found to express MMP-2, but pro-MMP-9 was detected in only one tissue sample and one cell line (U-2OS). The active form of MMP-9 was not expressed in any of the samples [10]. Our study also showed that the secreted protein level of MMP-9 was much lower than that of MMP-2 in our *in vitro* cell model. Otherwise, we found that MMP-2 was highly expressed in tumor specimens rather than normal bone and was positively correlated with the distal metastasis status of Taiwanese osteosarcoma patients. Our results echo previous findings which indicated that MMP-2 might be an important regulator of tumor metastasis in osteosarcomas.

Previous reports indicated that regulation of MMP expression is primarily at the transcriptional, posttranscriptional (modulation of mRNA stability), or protein level depending on the different activators or inhibitors [9, 43]. In osteosarcomas, we first showed that RESV can inhibit the MMP-2 protein level of tumor cells *in vitro* and *in vivo*. Moreover, promoter activity and *MMP-2* mRNA expression were also suppressed by RESV, indicating that regulation of RESV expression of MMP-2 might be partially at the transcriptional or posttranscriptional level.

An MMP-2 promoter revealed several regulatory elements, including p53, AP-1, Ets-1, C/EBP, CREB, PEA3, SP-1, and AP-2, which could be involved in regulating MMP-2 expression [44]. Our study indicated that regulation of MMP-2 by RESV is transcriptional and is mediated by CREB. CREB is a ubiquitously expressed transcription factor and is phosphorylated at Ser^133^ by cAMP-dependent protein kinase A, calcium^2+^-calmodulin, Akt, p38 MAPK, JNK, and other kinases [45-47] and subsequently increases its transcriptional activity by altering its association with the adaptor proteins, CBP/p300 histone acetylase. Our present study indicated that the p38 MAPK and JNK pathways are involved in RESV-mediated phosphorylation of CREB. Although a recent report indicated that phosphorylation of CREB at Ser^133^ by MAPK signaling does not promote strong recruitment of CBP or p300 [48], the effect of RESV on the interaction between CREB and CBP/p300 in osteosarcoma cells should be further elucidated in the future. Our findings implicating CREB phosphorylation in the regulation of MMP-2 in osteosarcomas are consistent with those in reports on melanomas [26] and ovarian cancer [33]. In addition, we also found that RESV can attenuate the DNA-binding activity of CREB in the MMP-2 promoter region.

The newly identified small noncoding RNAs, miRNAs, belong to a novel class of gene regulators that control posttranscriptional regulation of genes by binding to complementary sequences in the 3′UTRs of target mRNAs. Deregulated expression of miRNAs was reported in human cancers and may affect multiple steps during metastasis [49]. The ability of RESV to regulate miRNA expressions of miR-21, miR-155, and miR-520h was respectively investigated in prostate, leukemic, and lung cancer cells [50-52]. In addition to RESV-mediated transactivation inhibition of the *MMP-2* gene, we further investigated which miRNAs might be involved in RESV-mediated *MMP-2* gene expression and cell motility in osteosarcoma cells. Combining data from our miRNA screening profiles and miRNA target databases, we found that RESV treatment of osteosarcoma cells upregulated the expression of miR-328 which revealed the suppressive effects on MMP-2 and cell motility. Moreover, we also observed that the miR-328 expression level in osteosarcoma tissues was lower than that in normal bone tissues and was inversely correlated with the expression level of MMP-2 in osteosarcoma tissues. Until now, mir-328 has been recognized as a tumor suppressor or an oncogenic gene in different cancer types. For example, in the A431 human epithelial carcinoma cell line, miR-328 overexpression resulted in reduced cell adhesion, aggregation, and capillary formation by silencing CD44 [53]. It was also reported that miR-328 can decrease the chemoresistance of glioblastoma (GBM) and colorectal cancer (CRC) stem cells by downregulating ATP-binding cassette sub-family G member 2 (ABCG2) protein levels [54, 55]. In contrast, overexpression of miRNA-328 was reported to increase cell migration and to be associated with brain metastasis in non-small cell lung cancer [56].

Development of distal metastasis in osteosarcomas produces a high death rate. Herein, we first report that RESV showed antimetastasis activities via transcriptional and epigenetic regulation of MMP-2 by respectively altering the CREB-DNA-binding activity and miR-328 expression, which was initiated by inhibition of the p38 MAPK/JNK pathways (Figure 7D). In addition to MAPK signals, FAK activation was suppressed by RESV in osteosarcoma cells, and FAK was reported to regulate MMP-2 activity in endothelial cells [57]. However, the role of FAK in MMP-2 activation of osteosarcomas should be further investigated in future work. In conclusion, our study provides *in vitro*, *in vivo*, and clinical evidence that MMP-2 plays an important role in regulating metastasis of osteosarcomas and identifies the novel mechanisms that delineate how RESV can suppress the metastatic ability of osteosarcomas by targeting MMP-2 via transcriptional and epigenetic regulation. These results shed light on the mechanism of the antitumor effect of RESV and strongly support the development of clinical trials to determine whether RESV may be useful as a chemopreventive or chemotherapeutic agent in managing human osteosarcomas.

## MATERIALS AND METHODS

### Materials

RESV, SB203580, SP600125, and Ly294002 were purchased from Sigma-Aldrich (St Louis, MO). Cell culture materials and fetal bovine serum (FBS) were obtained from Gibco-BRL (Gaithersburg, MD). Antibodies specific for Bax, Bcl-2, MMP-2, TIMP-2, Ki-67, and β-actin were obtained from Santa Cruz Biotechnology (Santa Cruz, CA). Antibodies specific for unphosphorylated or phosphorylated forms of the corresponding ERK 1/2, JNK 1/2, p38, Akt, FAK, and CREB were purchased from Epitomic (Burlington, CA). The Mir-328 mimic and inhibitor were purchased from GenePharma (Shanghai, China). JNK, p38, and Akt dominant-negative mutants (DNs) were gifts from Dr. M. L. Kuo (National Taiwan University, Taipei, Taiwan). Unless otherwise specified, other chemicals used in this study were purchased from Sigma Chemical (St. Louis, MO). The primers used for amplification of the MMP-2 promoter gene region were as follows: forward primer, 5′-GGTACCCAGATCGCGAGAGAGGCAAGTAA-3′ and reverse primer, 5′-AAGCTTGGTTGGAGCCTGCTCCGCGGCG-3′. PCR-amplified DNA was digested with KpnI and HindIII and cloned into the pGL3-Luc-Basic reporter vector (Promega, Madison, MI).

### Cell culture

The human HOS, MNNG/HOS, and 143B osteosarcoma lines were purchased from American Type Culture Collection (Manassas, VA), while the MG-63, U2OS, and Saos-2 cell lines were purchased from the Food Industry Research and Development Institute (Hsinchu, Taiwan). HOS, MNNG/HOS, and 143B cells were cultured in α-minimum essential medium (MEM), and MG-63, U2OS, and Saos-2 cells were cultured in Dulbecco's modified Eagle medium (DMEM). Both media were supplemented with 10% FBS, 2 mM L-glutamine, 100 U/mL penicillin, and 100 μg/mL streptomycin at 37 °C in a humidified atmosphere containing 5% CO_2_.

### Cell viability assay

Osteosarcoma cell lines were grown to 80% confluence in 96-well plates, treated with RESV (25~100 μM) for 24 h, and then subjected to a cell viability assay (MTS assay; Promega) according to the manufacturer's instructions. Data were collected from three replicates.

### *In vitro* wound-closure assay

HOS, U2OS, and 143B cells (8 × 10^4^ cells/well) or MG-63 and Saos-2 cells (2 × 10^5^ cells/well) were plated in 24-well plates for 24 h, wounded by scratching with a pipette tip, then incubated with α-MEM or DMEM medium containing 0.5% FBS and treated with or without RESV (50~100 μM) for 24 h. Cells were photographed using a phase-contrast microscope (100×) as previously described [58].

### Transwell migration and invasion assays

Migration and invasion assays were performed as described previously.[52] Briefly, transwell migration assays used 2 × 10^5^ cells plated in a noncoated top chamber (24-well insert; pore size, 8 μm; Corning Costar, Corning, NY) and incubated for 24 h. The invasion assay used 10^5^ cells plated in a Matrigel (BD Biosciences, Bedford, MA)-coated top chamber and incubated for 24 h. In both assays, cells which had been pretreated for 24 h with RESV (25~100 μM) were plated in medium without serum or growth factors, and medium supplemented with serum was used as a chemoattractant in the lower chamber. After 24 h of incubation, cells that had not migrated or invaded through the pores were removed with a cotton swab. Cells on the lower surface of the membrane were fixed with methanol and stained with crystal violet. The number of cells migrating through or invading through the membrane was counted under a light microscope (×40, three random fields per well).

### Cell adhesion assay

After a 24-h treatment with 50 μM RESV, cells were washed and resuspended at a density of 2 × 10^5^ cells/mL in serum-containing α-MEM and then 100-μL aliquots were plated on 96-well dishes coated with 50 μg/mL collagen type I (BD Biosciences) or 20 μg/mL Matrigel and incubated for 40 min at 37 °C. Non-adherent cells were washed off with PBS, and adherent cells were fixed with 3.7% paraformaldehyde for 10 min at room temperature, followed by rinsing with PBS, and staining with 0.4% crystal violet (Sigma) for 10 min. After extensive rinsing, the dye was released from cells by adding 30% acetic acid, and the microtiter plates were read in a microplate reader at 590 nm.

### Immunofluorescence microscopy

HOS cells grown on coverslips were treated with RESV and fixed in 4% paraformaldehyde, permeabilized, and stained with Alexa Fluor® 488 Phalloidin (Life Technologies) to observe actin rearrangement or stained with a primary antibody against CREB (Epitomic, Burlingame, CA), followed by incubation with a rhodamine-conjugated secondary antibody (Santa Cruz Biotechnology, Santa Cruz, CA) to observe nuclear translocation. Slides were examined and photographed using a Zeiss Axiophot fluorescence microscope (Carl Zeiss Microimaging GmbH, Gottingen, Germany). Nuclei were counterstained with 4′,6-diamino-2-phenylindole (DAPI).

### Reverse-transcriptase polymerase chain reaction (RT-PCR)

Messenger (m)RNA was isolated and amplified as described previously [59]. Primer sequences are shown as supplementary data (Supplementary Table 1).

### Nuclear and cytosolic protein extraction

For nuclear and cytosolic protein extraction, protein extracts were prepared from HOS cells treated with 50 or 100 μM RESV using an NE-PER Cytoplasmic and Nuclear Protein extraction kit (Pierce Biotechnology, Rockford, IL).

### Western blot analysis

Protein lysates were prepared as described previously [59]. A Western blot analysis was performed with primary antibodies for FAK, p-FAK, MMP-2, TIMP-2, CREB, p-CREB, Akt, p-Akt, ERK, p-ERK, p38, p-p38, JNK, p-JNK, PCNA, or β-actin.

### Zymography

MMP-2, MMP-9, and uPA activities in conditional medium from osteosarcoma cells were measured using gelatin and casein zymography protease assays as described previously [60]. An appropriate volume of collected media was subjected to electrophoresis on 8% sodium dodecylsulfate polyacrylamide gel electrophoresis (SDS-PAGE) containing 0.1% gelatin for MMP2/9 activity determination or 2% w/v casein and 20μg/mL plasminogen for uPA activity determination. After electrophoresis, gels were washed with 2.5% Triton X-100 and incubated in reaction buffer (40 mM Tris-HCl at pH 8.0, 10 mM CaCl_2_, and 0.01% NaN_3_) for 12 h at 37 °C. The gel was then stained with Coomassie brilliant blue R-250.

### *In vivo* metastasis model

All animal work was performed in accordance with protocols approved by the Institutional Animal Care and Use Committee of Taipei Medical University. Male severe combined immunodeficient (SCID) mice, age-matched and 6~8 weeks old, were used in assays for tumor growth and lung metastasis in an orthotopic graft model. Luciferase-tagged MNNG/HOS cells (5 × 10^5^) were suspended in a 7:3 mixture of phosphate-buffered solution and Matrigel, and injected into the proximal tibia of SCID mice (seven mice per group). Nine days after tumor cell injection, the mice were orally fed resveratrol (40 or 100 mg/kg) or vehicle five times per week. The luciferase-based, noninvasive bioluminescent imaging and analysis were performed with the Xenogen IVIS-200 system (Xenogen, Alameda, CA). Twenty-four days after resveratrol treatment, animals were sacrificed, and tumor specimens were resected for immunohistochemical (IHC) staining.

### Patients and specimen preparation

The study protocol was approved by the Institutional Review Board of China Medical University Hospital, and all subjects gave informed written consent before enrollment. Specimens of tumor tissues or normal bone were obtained from patients who had been diagnosed with an osteosarcoma and had undergone surgical resection at China Medical University Hospital. Paraffin-embedded, formalin-fixed surgical specimens from all patients were collected for IHC staining.

### Tumor IHC

All tumor specimens were embedded in paraffin blocks and cut in 4-μm sections. All specimens were deparaffinized and immersed in 10 mM sodium citrate buffer (pH 6.0) in a microwave oven twice for 5 min to enhance antigen retrieval. After washing, slides were incubated with 0.3% H_2_O_2_ in methanol to quench endogenous peroxidase activity. Slides were washed with PBS and incubated with anti-Ki67, anti-MMP-2, and anti-mouse immunoglobulin G (IgG) antibodies for 2 h at room temperature. After washing in PBS, slides were developed with a VECTASTAIN ABC (avidin-biotin complex) peroxidase kit (Vector Laboratories, Burlingame, CA) and a 3,3,9-diaminobenzidine (DAB) peroxidase substrate kit (Vector Laboratories) according to the manufacturer's instructions. All specimens were deparaffinized and stained with hematoxylin and eosin (H&E) which was used as a light counterstain. IHC results of Ki67 were scored by taking into account the percentage of positive detection. IHC results of MMP-2 were classified into two groups according to the intensity and extent of staining We developed a MMP-2 image score by multiplying the intensity score (0~3 points) by the extent score (0~4 points) to represent the expression of MMP-2 in cancer tissues. Low and high expressions of MMP-2 were respectively defined as 0~6 and 8~12 points.

### Construction of MMP-2 promoter/reporter and MMP-2 3′UTR/reporter plasmids

The MMP-2 promoter was inserted into the pGL3-basic vector (Promega, Madison, WI) to generate the MMP-2 promoter/reporter plasmid. Site-directed mutagenesis of the CREB-binding site from the MMP-2 promoter was performed with a QuikChange Lightning Site-Directed Mutagenesis Kit (Agilent Technologies, Santa Clara, CA) according to recommendations in the instruction manual. The DNA sequence, GTGACGA, at position −205 was mutated to GTGGCAA, and the DNA sequence of the mutated promoter was verified with a sequence analysis.

The full-length sequence of the MMP-2 3′UTR is located at position 2295 of MMP-2 mRNA (NM_004530). We cloned the 3′ UTR region of human MMP-2 in a pGL2-basic vector (Promega, Madison, WI) downstream of the reporter gene. The predicted MMP-2-binding site of the miR-328 seed location in MMP-2 3′UTR is 817 which was identified by miRanda (http://www.microrna.org/microrna/home.do).

### Luciferase assay

HOS cells were seeded at a concentration of 5 × 10^4^ cells per well in 6-well cell culture plates. After 24 h of incubation, pGL3-basic (vector), pMMP-2-luciferase (Luc), or CREB-binding site mutant (mCREB) MMP-2-Luc were co-transfected with a β-galactosidase expression vector (pCH110) into cells using Turbofect (Fermentas, Carlsbad, CA). After 12 h of transfection, cells were treated with vehicle or RESV (25~100 μM) for 24 h. The luciferase activity was then determined using a luciferase assay kit. The value of luciferase activity was normalized to the transfection efficiency and monitored by β-galactosidase expression.

HOS cells were co-transfected with an miR-328 mimic or negative control and vector containing the MMP-2 3′UTR. The pRL-TK Renilla control vector (0.5 μg) (Promega) was also co-transfected as an internal control for transfection efficiency. GenMuteTM siRNA & DNA Transfection Reagent (SignaGen Laboratories, Ijamsville, MD) was used for this transfection process according to the manufacturer's instructions. Cells were harvested 48 h after transfection and analyzed for luciferase activity using the Dual-Luciferase Reporter Assay System (Promega).

### Lentiviral production and infection

pEZX-MR03-miR-328 (*Homo sapiens* microRNA miR-328 stem-loop expression clone, #HMIR0265), an HIV-based lentiviral vector containing the miR-328 precursor, and pEZX-MR03-control, which instead contains a scrambled sequence, were obtained from GeneCopoeia (Rockville, MD). The lentiviral vector and its packaging vectors were transfected into 293T packaging cells by calcium phosphate transfection. Briefly, 293T cells were split (10^6^) into 10-cm^2^ dishes 1 day before transfection. Then, cells were transfected with 10 μg pEZX-MR03-miR-328 or pEZX-MR03-control together with 10 μg of pCMVΔR8.91 (the packaging vector) and 1 μg of pMD.G (the envelope vector). Forty-eight hours later, lentivirus-containing medium was collected from transfection and spun down at 1500 rpm for 5 min to pelletize the cell debris, the supernatant was filtered through a 0.45-um filter, and target cells were infected with fresh lentivirus-containing medium (supplemented with 8 μg/ml polybrene) for 24 h.

### Chromatin immunoprecipitation (ChIP) analysis

A ChIP analysis was performed as described previously [59]. DNA immunoprecipitated with antibodies specific to CREB or the control, rabbit IgG, was purified and extracted using phenol-chloroform. Immunoprecipitated DNA was analyzed with a PCR or quantitative (q)PCR using specific primers which are described in supplementary data (Supplementary Table 1).

### TaqMan miRNA real-time RT-PCR

To determine the expression of miR-328 from osteosarcoma patients and cell lines, we used the TaqMan MicroRNA Assay kit (Applied Biosystems, Carlsbad, CA) following the manufacturer's protocol. Briefly, 10 ng of RNA from patient samples or cell lines was reverse-transcribed using 10 μl of an RT mixture containing dNTPs, RT, and RNase inhibitor and 3 μl of the respective primer. The mixture was incubated at 16 °C for 30 min, 42 °C for 30 min, and 85 °C for 5 min. Real-time PCRs were then carried out in a total volume of 20 μl of reaction mixture containing 2 μl of the RT product, 10 μl of 2 × Taqman universal PCR master mix, 7 μl of water, and 1 μl of the TagMan assay probe. All reactions, including the controls, were performed in triplicate. Relative expression of miRNAs was analyzed using the Ct method and was normalized to RNU6B expression.

### Statistical analysis

Values are presented as the mean ± SE. The statistical analysis was performed using Statistical Package for Social Science software, vers. 16 (SPSS, Chicago, IL). Data were analyzed using Student's *t*-test when two groups were compared. A one-way analysis of variance (ANOVA) followed by Tukey's post-hoc test was used to analyze three or more groups. Statistical analyses of clinicopathological data were performed by Chi-squared and Fisher's exact test. *p* values of < 0.05 were considered statistically significant.

## SUPPLEMENTARY MATERIAL, FIGURES, TABLES


