# Supplementary material for: Upregulation of miR-328 and inhibition of CREB-DNA-binding activity are critical for resveratrol-mediated suppression of matrix metalloproteinase-2 and subsequent metastatic ability in human osteosarcomas
Source: Oncotarget. 2014 Dec 30;6(5):2736–53. doi: 10.18632/oncotarget.3088 (PMC4413614; doi:10.18632/oncotarget.3088)
Supplement: Supplementary file 1 [file oncotarget-06-2736-s001.pdf]

Upregulation of miR-328 and inhibition of CREB-DNA-binding activity are critical for resveratrol-mediated suppression of matrix metalloproteinase-2 and subsequent metastatic ability in human osteosarcomas

Supplementary Material

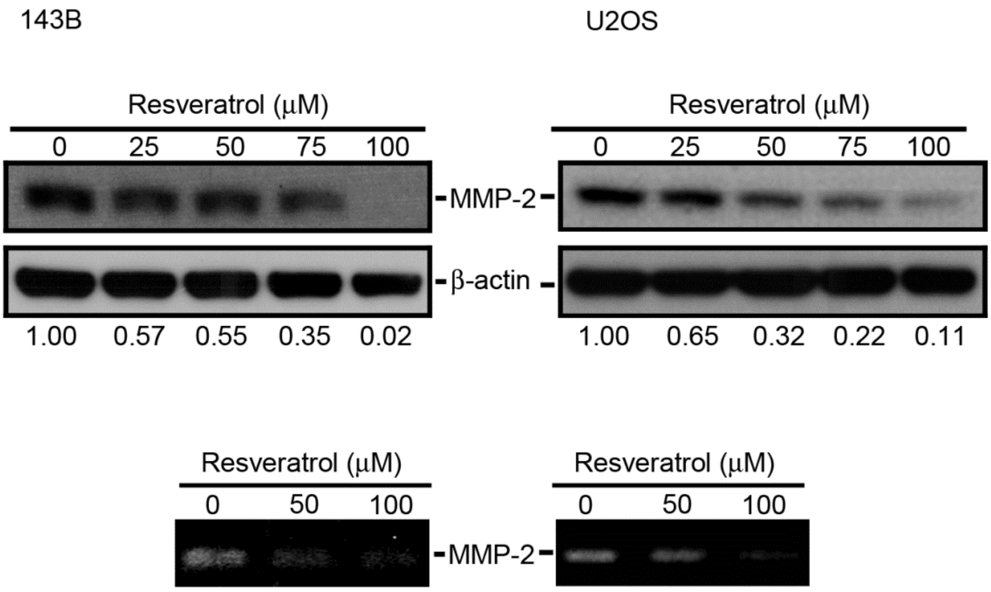

Figure S1: 143B or U2OS cells were treated with the vehicle or resveratrol (25~100 μM) for 24 h and then subjected to Western blot (upper panel) or zymography (lower panel) analysis. Quantitative MMP-2 protein levels were adjusted to the β-actin protein level.

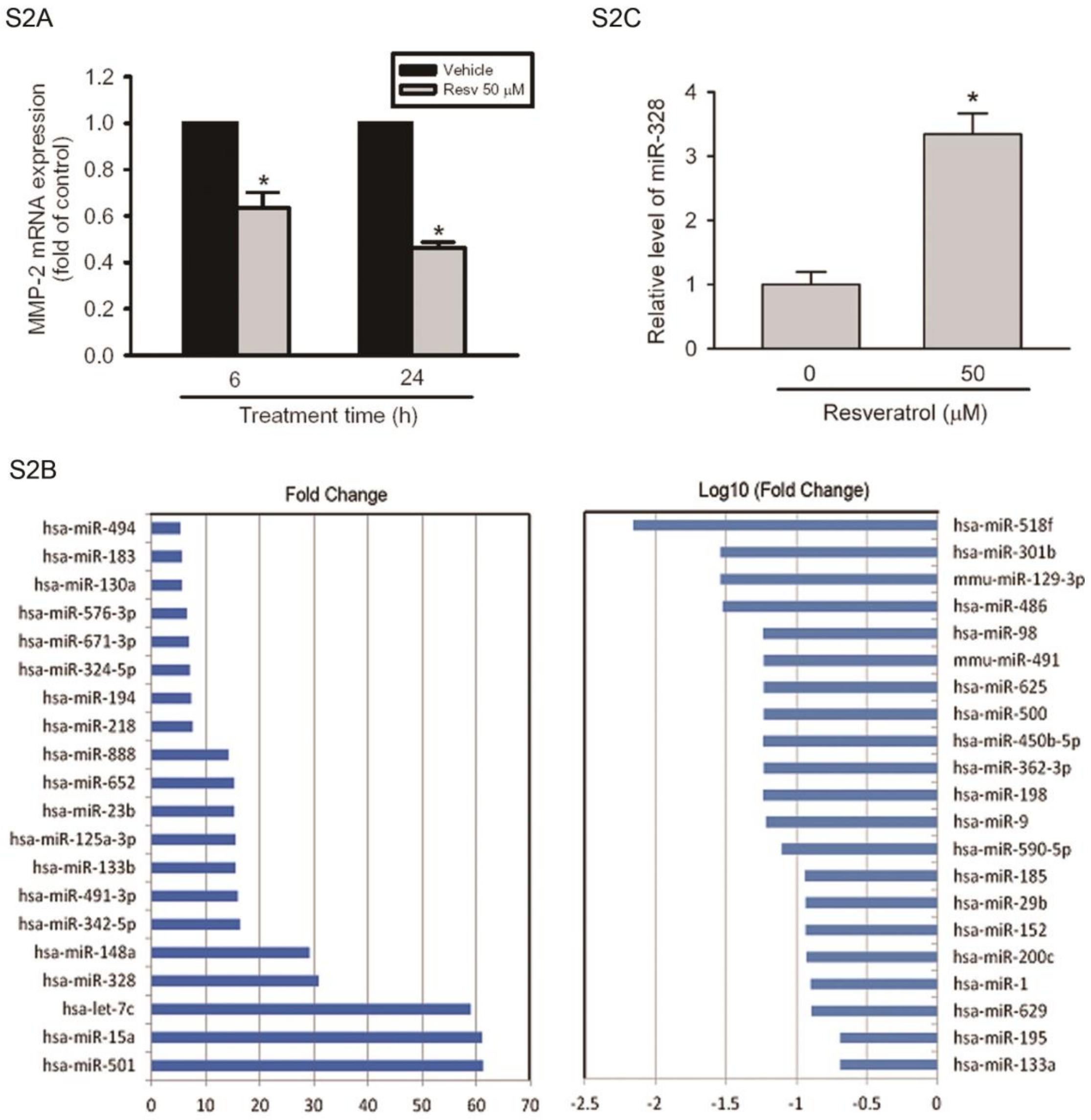

Figure S2: Representative miRNA expression after treatment of HOS cells with resveratrol (RESV). After treatment of osteosarcoma cells with RESV for 6 or 24 h, *MMP-2* mRNA was detected by a qPCR (A), and miRNA expression profiles were analyzed by a TaqMan Array Human MicroRNA. The top 20 up- or downregulated miRNAs are shown (B). Treatment of osteosarcoma cells with RESV for 6 h. miR-328 expression was detected by a qPCR (C) (* $p < 0.05$).

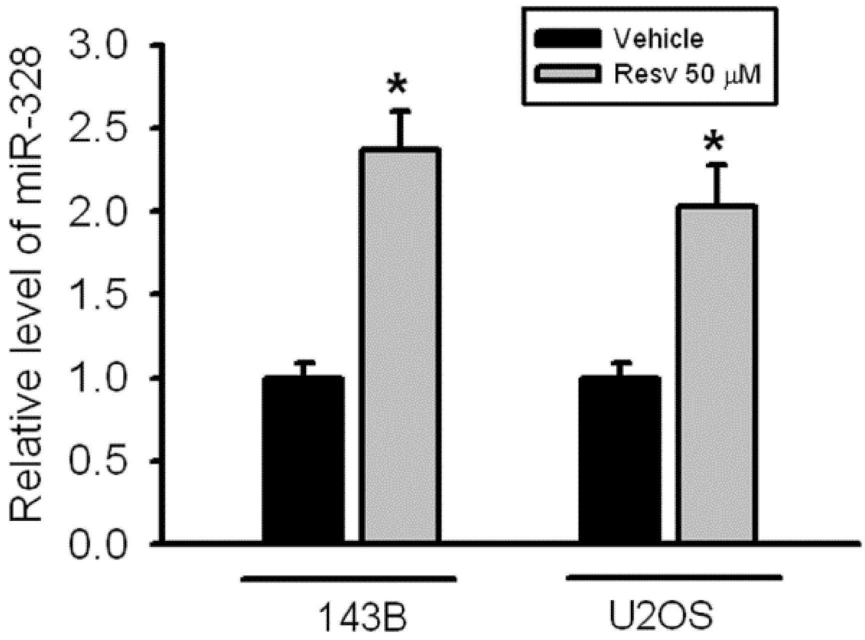

Figure S3: Treatment of osteosarcoma cells, 143B and U2OS, with resveratrol (RESV) for 6 h. miR-328 expression was detected by a qPCR (* $p < 0.05$).

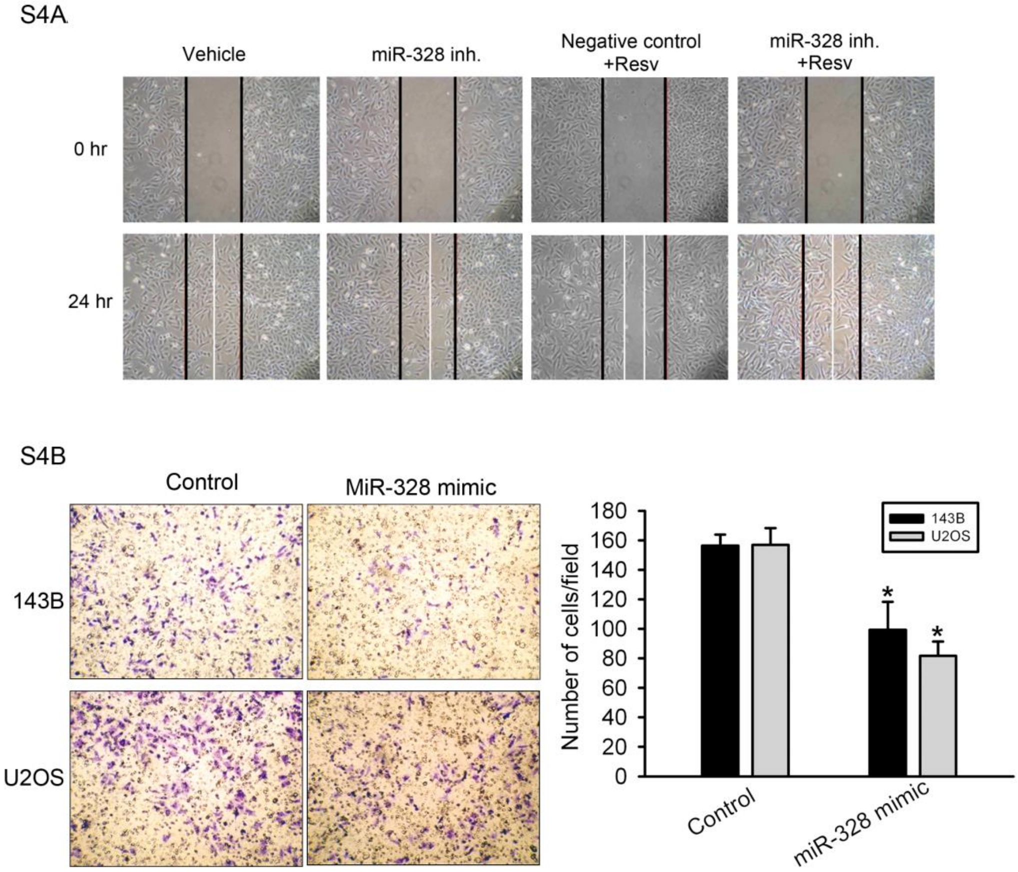

Figure S4: (A) HOS cells were transfected with an miR-328 inhibitor or negative control for 24 h followed by resveratrol (50 μM) treatment for an additional 24 h. The cell migratory ability was determined by a wound-closure assay. (B) 143 B or U2OS cells were transfected with an miR-328 mimic or non-targeting control, as indicated. The cell-invasion ability was determined by a Matrigel invasion assay.

Table S1: Primers list for RT-PCR, qPCR, and ChIP assay.

Primers used for RT- PCR

Gene	Species	Sequence (5’ to 3’)
MMP-2	Human	5’-GGCCCTGTCACTCCTGAGAT-3’ (forward)
		5’-GGCATCCAGGTTATCGGGGA-3’ (reverse)
GAPDH	Human	5’-CGGAGTCAACGGATTTGGTCGTAT-3’ (forward)
		5’-AGCCTTCTCCATGGTTGGTGAAGAC-3’ (reverse)

Primers used for ChIP

Gene	Species	Sequence (5’ to 3’)
MMP-2 promoter	Human	5’-GGGCCTAGAGCGACAGATGTTTC-3’ (forward)
CREB binding site		5’-AGCCTTCTCCATGGTTGGTGAAGAC-3’ (reverse)

Primers used for qPCR

Gene	Species	Sequence (5’ to 3’)
MiR-328	Human	5’-CUGGCCCUCUCUGCCCUUCCGU-3’
RNU6B	Human	5’-CGCAAGGATGACACGCAAATTCGTGAAGCGTTCCATATTTTT-3’
MMP-2	Human	5’-GATACCCCTTTGACGGTAAGGA-3’(forward)
		5’-CCTTCTCCCAAGGTCCATAGC-3’ (reverse)
